# Targeting inhibitors of apoptosis proteins suppresses medulloblastoma cell proliferation via G2/M phase arrest and attenuated neddylation of p21

**DOI:** 10.1002/cam4.1658

**Published:** 2018-07-09

**Authors:** Shu‐Mei Chen, Tzu‐Kang Lin, Yuan‐Yun Tseng, Chiao‐Hui Tu, Tai‐Ngar Lui, Shiang‐Fu Huang, Ling‐Ling Hsieh, Ying‐Ying Li

**Affiliations:** ^1^ Graduate Institute of Clinical Medical Sciences College of Medicine Chang Gung University Taoyuan Taiwan; ^2^ Department of Neurosurgery Wan Fang Hospital Taipei Medical University Taipei Taiwan; ^3^ Department of Neurosurgery School of Medicine Fu Jen Catholic University Hospital Fu Jen Catholic University New Taipei City Taiwan; ^4^ Department of Neurosurgery Shuang Ho Hospital Taipei Medical University Taipei Taiwan; ^5^ Department of Public Health College of Medicine Chang Gung University Taoyuan Taiwan; ^6^ Department of Otolaryngology‐Head and Neck Surgery Chang Gung Memorial Hospital Chang Gung University Taoyuan Taiwan; ^7^ Department of Medicine University of Miami Miller School of Medicine Miami Florida

**Keywords:** cell cycle, inhibitors of apoptosis proteins, medulloblastoma, neddylation, p21

## Abstract

Medulloblastoma (MB) is the most common type of malignant childhood brain tumor. We previously showed that inhibitors of apoptosis proteins (IAP) small‐molecule inhibitors (LCL161 or LBW242) combined with chemotherapy have synergistic antiproliferative effects on MB cells. The synergistic antitumor effects of combination treatments happen through induction of autophagy and caspase‐3/7‐activated apoptosis. Here, we investigated the effects of IAP inhibitors or silencing IAP on cell cycle regulation. We discovered that treatment with IAP inhibitors or their combination with conventional chemotherapy (vincristine or cisplatin), as well as RNAi knockdown of cIAP1/2 or XIAP arrested MB cells in the G2/M phase through downregulation of cyclin B1‐CDK1 and cyclin A‐CDK1/2. Among these three IAPs, only silencing cIAP1 expression enhanced p21 dependent‐G2/M phase accumulation. IAP inhibitors reduced cIAP1 expression and increased p21 expression in time course experiments. Furthermore, cIAP1 can govern p21 proteasomal degradation via neddylation in lieu of ubiquitination. Inhibition of IAPs significantly abrogated cIAP1‐mediated p21 degradation. We also observed an inverse correlation between nuclear cIAP1 and nuclear p21 expressions in MB tumor tissues. These findings provide new mechanistic evidence of the influence of IAP inhibitors on MB cell proliferation through disruption of the cell cycle.

## 1 INTRODUCTION

Medulloblastoma (MB), an embryonic tumor arising in the cerebellum, comprises 15%‐30% of all pediatric central nervous system tumors and is the most common malignant primary brain tumor in children.^1^, ^2^, ^3^ Even with multimodal strategies including surgery, radiation, and chemotherapy, tumor recurrence is frequent and most patients eventually succumb to progressive disease.^4^, ^5^, ^6^, ^7^, ^8^, ^9^ Conventional chemotherapy alone can effectively eliminate nonmetastatic MB, yet it is not sufficient to treat metastatic MB.^10^ Additionally, conventional chemotherapy allows to reduce the dose of radiation therapy; however, the inferior outcome of chemo‐radiation therapy is due to treatment interruption attributed to myelosuppression.^11^ Hence, developing new treatments is an urgent need for MB patients.

Our previous study illustrated that inhibitors of apoptosis proteins (IAP) are highly expressed in MB cell lines and tissues and even higher in MB cancer stem‐like cells.^12^ IAP inhibitors (LCL161 or LBW242) in combination with conventional chemotherapeutic agents (i.e., vincristine and cisplatin) exhibit synergistic effects on MB cell proliferation and elicit concomitant type I (apoptotic) and type II (autophagic) cell death through activation of caspase‐3/7 and autophagic flux in MB cells.^12^

Inhibitors of apoptosis proteins are highly conserved proteins known for the regulation of caspases. The three best‐characterized IAPs include X‐linked IAP (XIAP), cellular IAP1 (cIAP1), and cellular IAP2 (cIAP2). They have conserved regions including baculovirus IAP repeats (BIRs) and the RING (Really Interesting New Gene) domains.^13^ The BIR domain is responsible for protein‐protein interaction with caspases and hence suppresses mitochondria‐dependent and independent apoptosis.^14^, ^15^, ^16^ The RING domain of IAPs acts as an E3 ligase, leading to ubiquitination of IAPs themselves and their client proteins, such as caspases. Recent studies discovered that neuronal precursor cell‐expressed developmentally downregulated protein 8 (NEDD8), a ubiquitin‐like protein,^17^ is activated by IAPs in the regulation of apoptosis through neddylation of caspases.^18^, ^19^, ^20^ IAPs function in ways beyond inhibition of apoptotic proteins. They are also implicated in inflammatory signaling, cell immunity, mitogenic kinase signaling, proliferation, cell invasion, and cell cycle.^21^, ^22^, ^23^

It remains unclear how IAPs regulate the cell cycle. The typical example is survivin, an IAP that connects anti‐apoptotic pathways and the cell cycle. Survivin partially suppresses caspase cascade triggered by Fas, Bax, and the anticancer drug etoposide,^24^ and interacts with microtubules located in the mitotic spindle when the cells are in the G2/M phase transition. Disrupting survivin‐microtubule interaction increases caspase‐3 activity in the G2/M phase.^25^

With respect to distribution of IAPs in mammalian cells, cIAP1 is predominantly nuclear, while XIAP is predominantly cytoplasmic and cIAP2 is both nuclear and cytoplasmic.^26^, ^27^ One study reported that the BIR domain of nuclear cIAP1 can interact with the DNA binding domain of transcription factor E2F1, and in turn stimulate E2F1 transcriptional activity, which controls the G1/S phase transition in human hematopoietic cells.^28^

In this study, we found that IAP inhibitors (LCL161 or LBW242) alone or in combination with a chemotherapeutic agent (vincristine or cisplatin) as well as XIAP or cIAP1/2 ablation using siRNA can inhibit the proliferation of MB cells (DAOY and D283MED) by inducing G2/M phase arrest. G2/M phase arrest corresponded to downregulated cyclin A, cyclin B1, cyclin‐dependent kinase 1 (CDK1), and cyclin‐dependent kinase 2 (CDK2) expression. Furthermore, silencing cIAP1 expression was able to upregulate cyclin‐dependent kinase inhibitor (CKI) p21 by impairing its neddylation‐mediated proteasomal degradation. Hence, these findings demonstrated that blockade of IAPs not only enhances cell death but also perturbs cell cycle through previously unknown mechanisms.

## 2 MATERIALS AND METHODS

### 2.1 Tissue array and immunohistochemical (IHC) staining

The MB tissue array (BC17012b) was purchased from US Biomax, Inc. (Rockville, MD, USA). The method of IHC staining has been described in previous study.^12^ Primary antibodies against XIAP, cIAP1, cIAP2, and p21 were purchased from Proteintech, Santa Cruz Biotechnology, R&D systems, and Santa Cruz Biotechnology, respectively. The method of scoring cIAP1, cIAP2, and p21 expression was based on the criteria of H‐score proposed by K.S. McCarty.^29^

### 2.2 Cell lines

Medulloblastoma (MB) cell lines DAOY and D283MED and normal fibroblast cell line BJ were purchased from the American Type Culture Collection (ATCC). DAOY, D283MED, and BJ cells were cultured in minimum essential medium (MEM; Life Technologies), which has been mentioned in previous studies.^12^ The human astrocyte‐hippocampal (HA‐h, ScienCell Research Laboratories) cell line was kindly provided by Dr. Ruei‐Ming Chen (Taipei Medical University, Taiwan), and cultured in Astrocyte Medium (ScienCell Research Laboratories).

### 2.3 Reagents

The IAP inhibitors LCL161 and LBW242 were obtained from Active Biochemicals Co., Limited (Hongkong, China) and Novartis Pharmaceuticals (Basel, Switzerland). Vincristine (Teva Pharmaceuticals, Petah Tikva, Israel) and cisplatin (Teva Pharmaceuticals, Petah Tikva, Israel) were obtained from Wan Fang Hospital pharmacy in Taipei, Taiwan. MG‐132 (Selleckchem, Houston, TX, USA), cycloheximide (CHX; Sigma‐Aldrich, St. Louis, Missouri‎, USA), and MLN4924 (TargetMol, Boston, MA, USA) were utilized to suppress proteasome activity, protein synthesis, and neddylation, respectively.

### 2.4 Cell viability assay

This assay was carried out using thiazolyl blue tetrazolium bromide (MTT; Sigma), as described in previous studies.^12^ Cell viability was calculated using the formula: (OD of experimental well/OD of control well) × 100%.

### 2.5 Immunoblotting

Antibody against XIAP, cIAP2, p21, or p53 was purchased from Cell Signaling Technology (‎Danvers, MA, USA); antibody for detection of cIAP1, cIAP1/2, p27, p16, GAPDH, cyclin A, or cyclin B1 was purchased from Santa Cruz Biotechnology (Dallas, TX, USA); antibodies against CDK1 and CDK2 were bought from Millipore (Burlington, MA, USA) and Upstate Biotechnology, respectively. The blotting membrane was developed using enhanced chemiluminescence (ECL) substrate (Millipore), and analyzed by densitometry and Image J (National Institutes of Health, USA).

### 2.6 Cell cycle analysis

MB cells were harvested following treatment with reagents. Cells were fixed with 70% ethanol, and then stored at −20°C overnight. Subsequent to equilibrating to room temperature, the cells were permeabilized with PBS containing 0.5% Triton X‐100 and 0.05% RNAse followed by staining with 50 μg/mL propidium iodide (PI; Sigma) at 4°C for 30 minutes. Finally, DNA content was detected by fluorescence‐activated cell sorting (FACS; Beckman Coulter Epics XL, Brea, CA, USA), and data were analyzed by EXPO32 ADC software (Beckman‐Coulter, USA).

### 2.7 Immunoprecipitation

To observe ubiquitination or neddylation of p21, anti‐p21 antibody (Cell Signaling Technology) and Meg‐Beads‐Protein G (TOOLs) were added into total cell lysates collected from MB cells after transfected with plasmid overexpressing ubiquitin‐hemagglutinin (HA; Addgene, Cambridge, MA, USA) or NEDD8‐HA (Addgene) and treated with MG‐132 for 6 hours. Thereafter, immunoprecipitates were analyzed by immunoblotting with anti‐HA antibody (Sigma).

### 2.8 Transfection of siRNA

Small interfering RNA (siRNA) specific to p21, cIAP1, cIAP2, cIAP1/2, or XIAP, and nontargeting (NT) siRNA were purchased from TOOLS. Prior to transfection with siRNA (10 nmol/L), MB cells were cultured in antibiotic‐free media for 24 hours. Transfection was carried out in antibiotic‐free media using INTERFERin transfection reagent (Polyplus) per manufacturer's instruction.

### 2.9 RNA analysis

Total RNA was extracted using trizole reagent (Easypure Total RNA Reagent) and converted into cDNA product using iScript cDNA Synthesis Kit (BIO‐RAD, Hercules, CA, USA). The cDNA product was mixed with PCR Master Mix reagent (Promega, Madison, WI, USA) and PCR primers, and then subjected to 1% agarose gel electrophoresis. The target gene expression was normalized by GAPDH. Primer sequences for p21 are 5′‐GCGATGGAACTTCGACTTTGT‐3′ (forward) and 5′‐GGGCTTCCTCTTGGA‐GAAGAT‐3′ (reverse); primer sequences for cIAP1 are 5′‐CCTGTGGTTAAATCTGCCTTG‐3′ (forward) and 5′‐CAATTCGGCACCATAACTCTG‐3′ (reverse); primer sequences for cIAP2 are 5′‐AAGTTCCTACCACTGTGCAATG‐3′ (forward) and 5′‐CAAGTAGATGAGGGTAA‐CTGGC‐3′ (reverse); primer sequences for GAPDH are 5′‐TGAAGGTCGGAGTCA‐ACGGATTTGGT‐3′ (forward) and 5′‐CATGTGGGCCATGAGGTCCACCAC‐3′ (reverse).

### 2.10 Immunofluorescence

Cells were grown on chamber slides for at least 16 hours, fixed in 4% paraformaldehyde for 15 minutes at room temperature, permeabilized with 0.1% Triton X‐100 for 10  minutes, and then incubated with blocking buffer (PBS containing 0.3% Triton X‐100 and 3% BSA) at room temperature for 1 hour. Afterward, they were incubated with antibodies against p21 (Cell Signaling Technology), cIAP1 (Santa Cruz Biotechnology), and cIAP2 (ABclonal) at 4°C overnight. Cells were then washed and incubated with fluorophore‐conjugated secondary antibodies (goat anti‐rabbit IgG TRITC; goat anti‐mouse IgG Alexa Fluor 488) for 1 hour at room temperature. Finally, cells were washed, air‐dried, and covered with DAPI‐containing Citifluor mounting medium.

### 2.11 Statistical analysis

Statistical analysis was carried out using Microsoft Excel software and SigmaPlot 10.0 (Systat Software Inc, Chicago, IL, USA). Statistical significance was based on student's *t* test and the *P‐*value <0.05 represents statistical significance.

## 3 RESULTS

### 3.1 IAP inhibitors alone and in combination with conventional chemotherapy display anti‐proliferative effect on MB cells with high levels of XIAP and cIAP1/2

**Figure 1:**
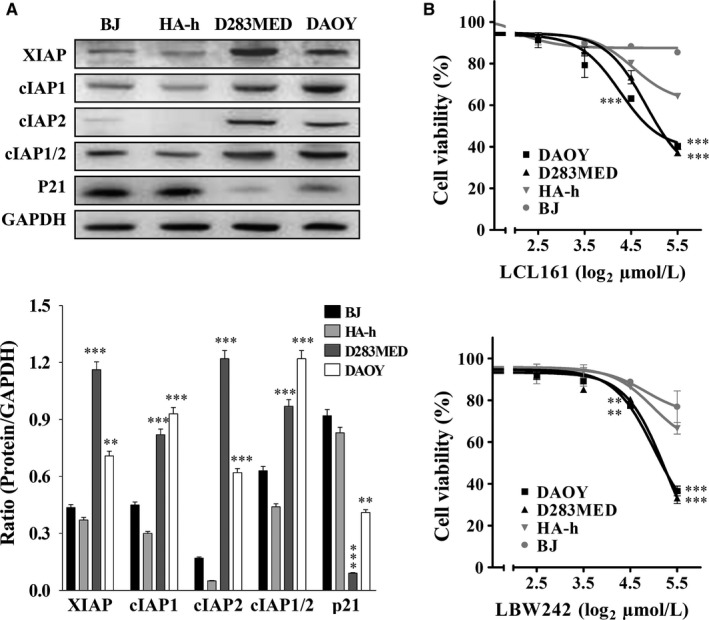
High Levels of IAPs in MB Cells Correspond to Sensitivity to IAP Inhibitors LBW242 or LCL161. A, The levels of IAPs and p21 in MB cell lines DAOY, D283MED and normal controls HA‐h and BJ were determined by immunoblotting. Their levels in MB cells were quantitated, normalized by GAPDH, presented in a bar graph, and compared to those in HA‐h cells. B, DAOY, D283MED, HA‐h, and BJ cell lines were treated with LBW242 or LCL161 at different concentrations (0, 5, 10, 20, and 40 μmol/L) and DMSO (control) for 72 h. Cell viability was determined by MTT assay. Data are represented as mean ± SEM of three independent experiments (*P <* *0.05, **P <* *0.01, and ***P <* *0.005)

We previously found lower levels of XIAP and cIAP1/2 in normal human astrocytes (HA‐h) than in MB cells (DAOY and D283MED).^12^ To confirm whether cIAP1, cIAP2, or both were highly expressed in MB cell lines, we assessed their expression including XIAP by immunoblotting. The result revealed that DAOY and D283MED cells expressed higher levels of XIAP, and cIAP1 or/and cIAP2 compared to HA‐h and immortalized fibroblasts (BJ; Figure 1A). Additionally, 30 μmol/L of IAP inhibitors (LCL161 or LBW242) inhibited 50% of proliferation activities in MB cells but only mildly slowed BJ or HA‐h cell proliferation (Figure 1B). Treatment with a low dose of LCL161 or LBW242 (10 μmol/L) significantly lowered the IC50 value of cisplatin or vincristine in MB cells but not in BJ or HA‐h cells (Table 1), and drastically enhanced cisplatin‐ or vincristine‐induced apoptosis in MB cells (Table 2). This result suggested that sensitivity to IAP inhibitors correlates with XIAP, cIAP1, and cIAP2 expression in MB cells.

**Table 1:** IC50 of chemotherapeutic agent or in combination with IAP inhibitor for DAOY, D283MED, BJ, and HA‐h cells

	DAOY	D283MED	BJ	HA‐h
Vincristine	5.5 ± 0.61 nmol/L	5.7 ± 0.47 nmol/L	>5 nmol/L	>20 nmol/L
Vincristine + LCL16	1.1 ± 0.20 nmol/L	2.4 ± 0.11 nmol/L	>5 nmol/L	>20 nmol/L
Vincristine + LBW242	1.3 ± 0.18 nmol/L	1.8 ± 0.32 nmol/L	>10 nmol/L	>20 nmol/L
Cisplatin	1.8 ± 0.10 μmol/L	1.2 ± 0.25 μmol/L	>5 μmol/L	>5 μmol/L
Cisplatin + LCL161	0.3 ± 0.13 μmol/L	0.6 ± 0.02 μmol/L	>5 μmol/L	>5 μmol/L
Cisplatin + LBW242	0.43 ± 0.02 μmol/L	0.5 ± 0.03 μmol/L	>10 nmol/L	>5 μmol/L

**Table 2:** Proportions of apoptotic DAOY and D283MED cells after treatment with chemotherapy or in combination with IAP inhibitor

Cell line	Treatment	Apoptosis (%)^a^	*P*‐value
DAOY	Control	6.6 ± 3.2%	
	LCL161	17.2 ± 2.5%	0.0106
	LBW242	22.6 ± 1.2%	0.0013
	Vincristine	6.0 ± 2.1%	
	Vincristine + LCL161	42.1 ± 0.2%	>0.0001
	Vincristine + LBW242	51.2 ± 3.8%	>0.0001
	Cisplatin	13.3 ± 2.4%	
	Cisplatin + LCL161	34.0 ± 8.0%	0.0127
	Cisplatin + LBW242	54.7 ± 1.0%	>0.0001
D283MED	Control	11.3 ± 0.2%	
	LCL161	14.0 ± 0.4%	0.0005
	LBW242	20.1 ± 0.3%	>0.0001
	Vincristine	34.4 ± 2.4%	
	Vincristine + LCL161	49.7 ± 2.0%	0.0011
	Vincristine + LBW242	59.0 ± 3.2%	0.0004
	Cisplatin	55.3 ± 3.5%	
	Cisplatin + LCL161	78.7 ± 2.1%	0.0006
	Cisplatin + LBW242	77.3 ± 0.6%	0.0004

a Apoptosis was detected by Annexin V/PI and apoptotic proportion was quantitated by FACS based on Annexin V‐positive population.

### 3.2 Treatment with IAP inhibitors interrupts cell cycle in MB cells

**Figure 2:**
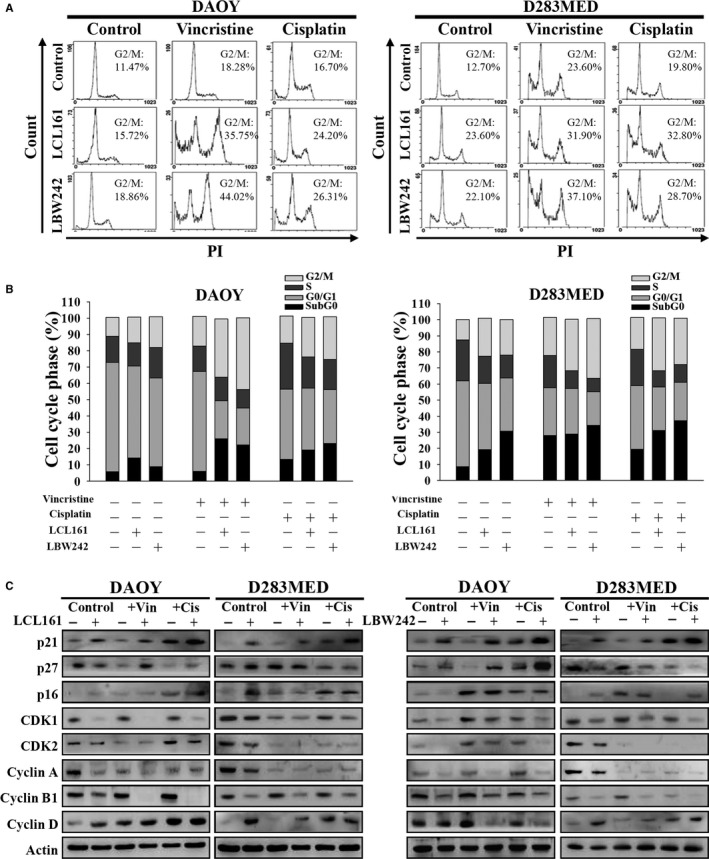
Treatment with IAP Antagonists or in Combination with Chemotherapeutics Induces G2/M Phase Arrest in MB Cells. A, MB cells were treated with DMSO (control), vincristine (1.25 nmol/L for DAOY and 2.5 nmol/L for D283MED), or cisplatin (0.31 μmol/L for DAOY and 0.625 μmol/L for D283MED), or in combination with LCL161 (10 μmol/L) or LBW242 (10 μmol/L) for 72 h. Thereafter, cells were harvested and their DNA contents were analyzed by FACS. B, The proportion of each cell cycle compartment was shown in bar graphs. C, The levels of cell cycle‐related proteins were assessed by immunoblotting subsequent to treatment with cisplatin or vincristine in the presence or absence of IAP inhibitors for 72 h

As our previous results demonstrated that IAP inhibitors suppress cell proliferation and induce cell apoptosis in MB cells, we next investigated whether these inhibitors reduce MB cell proliferation by disturbing the cell cycle. DAOY and D283MED cells were treated with LCL161 or LBW242 (10 μmol/L) and their DNA content was analyzed by propidium iodide (PI) and flow cytometry (FACS). The results indicated that treatment with IAP inhibitors slightly increased accumulation of sub‐G0 and G2/M transition in MB cells (Figure 2A,B). Combination of IAP inhibitors (10 μmol/L) and IC50 doses of vincristine (1.25 nmol/L for DAOY and 2.5 nmol/L for D283MED) or IC50 doses of cisplatin (0.31 μmol/L for DAOY and 0.62 μmol/L for D283MED) increased the proportion of cells in sub‐G0 and G2/M phase relative to IAP inhibitors (LCL161or LBW242) alone (Figure 2A,B). Compared to vincristine alone, vincristine combined with IAP inhibitors increased 5%‐15% arrest in sub‐G0 phase and 8%‐30% arrest in G2/M phase. Moreover, combination of IAP inhibitors and cisplatin could augment 3.5‐23% sub‐G0 arrest and 9%‐12% G2/M arrest relative to cisplatin alone (Figure 2B). These data indicated that IAP antagonism alone or in combination with chemotherapy decreases cell proliferation via cell cycle arrest.

### 3.3 IAP inhibitor induces G2/M phase arrest through downregulation of cyclin B1‐CDK1 and cyclin A‐CDK1/2 and upregulation of p21

We examined the expression of G2/M transition‐related proteins including CDK1, CDK2, cyclin A, and cyclin B1 by immunoblotting. As shown in Figure 2C, either IAP antagonist (LCL161 or LBW242) or as add‐on treatment to vincristine or cisplatin decreased the protein levels of CDK1, CDK2, cyclin A and cyclin B1. Downregulation of cyclin B1‐CDK1 expression was more evident than that of cyclin A‐CDK2 or cyclin A‐CDK1 in MB cells following treatment with IAP antagonist or in combination with vincristine or cisplatin. These data are in accordance with other studies showing that attenuation of the cyclin B1‐CDK1 complex, which is critical for entry into mitotic phase, can be seen in cancer cells arresting in the G2/M transition.^30^, ^31^

To understand the mechanism leading to downregulation of cyclin B1‐CDK1, several vital cyclins and CDK inhibitors (CKIs) in the progression of the cell cycle, including p16, p21, and p27 were analyzed by immunoblotting. MB cells treated with IAP inhibitors combined with or without vincristine or cisplatin increased p21 expression (Figure 2C). Consistent with this notion, the levels of IAPs including XIAP, cIAP1, and cIAP2 inversely correlated with p21 expression in MB cells, normal fibroblasts, and astrocytes (Figure 1A). Hence, IAPs are involved in cell cycle progression by suppressing p21 expression.

### 3.4 Like IAP antagonism, ablation of XIAP or cIAP1/2 results in cell cycle arrest in G2/M

**Figure 3:**
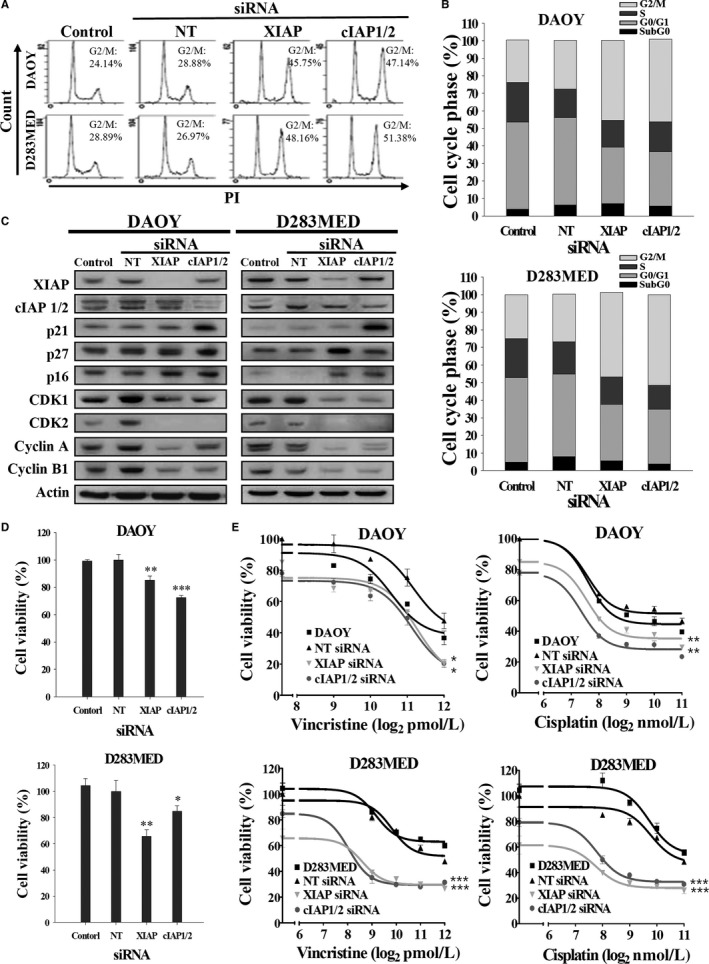
Knockdown of XIAP or cIAP1/2 Expression Disrupts Cell Cycle, and Renders MB Cells Sensitive to Conventional Chemotherapy. A, MB cells were transfected with siRNAs against XIAP or cIAP1/2, or nontargeting (NT) siRNA for 72 h. Afterward, the cell cycle was analyzed by FACS. B, The proportion of each cell cycle compartment is shown in bar graphs. C, Total cell lysates were subjected to immunoblotting for detecting cell cycle‐related proteins. D, Cell viability was determined by MTT assay. E, MB transfectants were treated with vincristine or cisplatin at different concentrations for 72 h. The viability curves were determined by MTT assay. Data were represented as mean ± SEM of three independent experiments (*P <* *0.05, **P <* *0.01, and ***P <* *0.005)

To verify whether IAPs are implicated in regulation of cell cycle and p21 expression, we silenced XIAP and cIAP1/2 expression separately with specific siRNAs. Knockdown efficiency of siRNAs has been confirmed by immunoblotting (Figure 3C). The control group was nontargeting (NT) siRNA. After transfected with siRNAs against XIAP or cIAP1/2 for 72 hours, both DAOY and D283MED cells displayed around 10%‐30% decreased growth rate and a drastic increase in G2/M phase (NT siRNA vs. XIAP or cIAP1/2 siRNA is 25% vs. 43%‐45%; Figure 3A‐C). Even knockdown of cIAP1 or cIAP2 in DAOY and D283MED cells increased arrest by 8%‐11% in the G2/M phase compared to the control group (Figure S1). Similar to treatment with IAP antagonists, ablation of either XIAP or cIAP1/2 expression decreased the levels of cyclin A, cyclin B1, CDK1, and CDK2 (Figure 3C and Figure S1). Only silencing cIAP1/2 expression in both DAOY and D283MED cells resulted in elevated p21 expression (Figure 3C).

Silencing XIAP or cIAP1/2 inhibited proliferation and sensitized MB cells to vincristine and cisplatin (Figure 3D,E, and Table 3). Silencing XIAP or cIAP1/2 also increased G2/M phase arrest in DAOY and D283MED cells (Figure S2). Ablation of cIAP1/2 combined with vincristine or cisplatin treatment not only reduced viability in MB cells compared to vincristine or cisplatin alone but also significantly switched the cell cycle toward the G2/M phase and enhanced chemotherapy‐induced G2/M phase arrest in DAOY and D283MED cells, while ablation of XIAP combined with chemotherapy yielded less G2/M phase accumulation in DAOY cells (Figure S2). Silencing XIAP or cIAP1/2 significantly promoted vincristine‐induced sub‐G0 phase arrest in both cell lines (Figure S2). Altogether, these data suggested that knockdown of cIAP1/2 expression switches cell cycle toward G2/M transition in the presence or absence of chemotherapeutic agent through upregulation of p21 expression as well as IAP inhibitors treatment.

**Table 3:** IC50 of chemotherapeutic agent or combined with knockdown of cIAP1 or cIAP2 for DAOY and D283MED cells

Cell line	Treatment	IC50	*P*‐value^a^
DAOY	Vincristine	3.47 ± 0.28 nmol/L	
	NT siRNA + Vincristine	4.78 ± 0.46 nmol/L	
	XIAP siRNA + Vincristine	2.76 ± 0.09 nmol/L	0.0259
	cIAP1/2 siRNA + Vincristine	2.43 ± 0.33 nmol/L	0.0278
	Cisplatin	0.74 ± 0.06 μmol/L	
	NT siRNA + Cisplatin	1.31 ± 0.13 μmol/L	
	XIAP siRNA + Cisplatin	0.28 ± 0.01 μmol/L	0.0079
	cIAP1/2 siRNA + Cisplatin	0.21 ± 0.01 μmol/L	0.0070
D283MED	Vincristine	>5 nmol/L	
	NT siRNA + Vincristine	4.44 ± 0.29 nmol/L	
	XIAP siRNA + Vincristine	0.37 ± 0.05 nmol/L	0.0026
	cIAP1/2 siRNA + Vincristine	0.44 ± 0.05 nmol/L	0.0027
	Cisplatin	>2.5 μmol/L	
	NT siRNA + Cisplatin	2.39 ± 0.13 μmol/L	
	XIAP siRNA + Cisplatin	0.17 ± 0.10 μmol/L	0.0027
	cIAP1/2 siRNA + Cisplatin	0.31 ± 0.03 μmol/L	0.0021

a *P*‐value is calculated after comparing to nontargeting siRNA (NT siRNA) treatment.

### 3.5 LBW242 treatment increases p21 protein expression via inhibition of cIAP1 in MB cells

**Figure 4:**
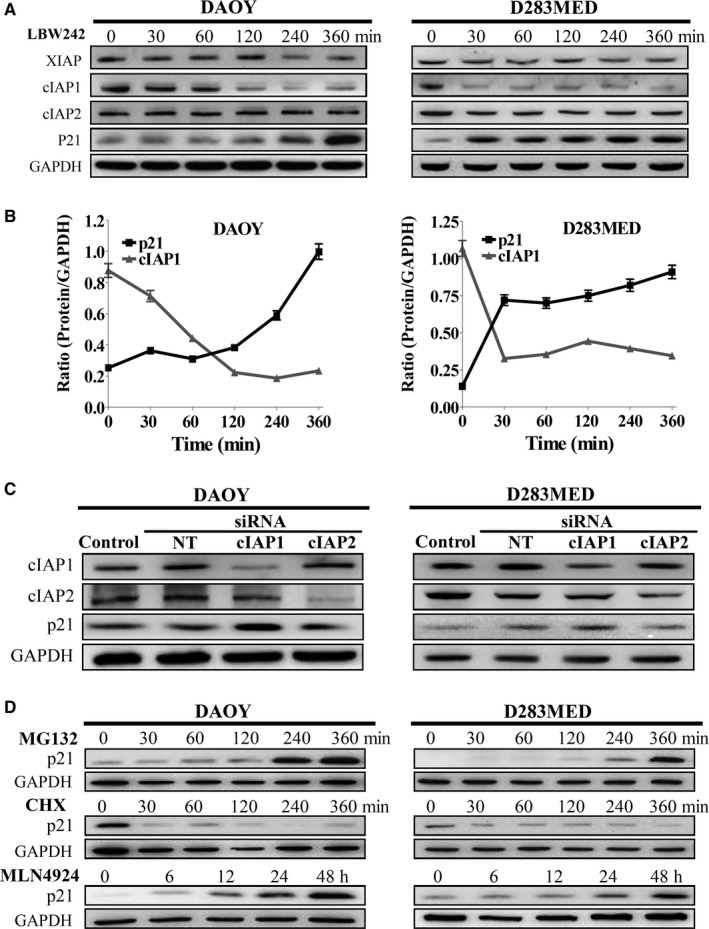
IAPs Inhibition Increases the Protein Levels of p21, which are Governed by Proteasomal Degradation. A, DAOY and D283MED cells were incubated with LBW242 (10 μmol/L) for 0‐360 min. Total cell lysates were collected at different time points and then subjected to immunoblotting. B, The protein levels of cIAP1 and p21 were quantitated, normalized by the levels of GAPDH, and shown in the curve graphs. C, MB cells were transfected with siRNAs against cIAP1 or cIAP2, and nontargeting (NT) siRNA, and protein levels of cIAP1, cIAP2, and p21 were detected by immunoblotting. D, MB cells were treated with MG‐132, CHX, and MNL4924 to verify p21 protein degradation. The levels of p21 were analyzed by immunoblotting

As our data have shown that IAPs inhibition enhances p21 expression in DAOY and d283MED cells, we next verified that increased p21 expression is through transcriptional regulation or protein degradation. DAOY and D283MED cells were treated with LBW242 (10 μmol/L), and protein levels of XIAP, cIAP1, and cIAP2 were detected at different time intervals. LBW242 effectively inhibited cIAP1 rather than cIAP2 or XIAP expression in 60 minutes (Figure 4A,B). Among these IAPs, only cIAP1 expression inversely correlated with p21 expression. To confirm this relationship, cIAP1 and cIAP2 in MB cells were separately silenced with specific siRNAs. The result showed the protein levels of p21 were increased by silencing cIAP1 but not silencing cIAP2 (Figure 4C). However, neither LBW242 treatment nor cIAP1/2 ablation altered p21 transcriptional levels (Figures S3 and S4). Taken together, these results revealed that LBW242 enhances p21 expression via attenuation of cIAP1.

**Figure 5:**
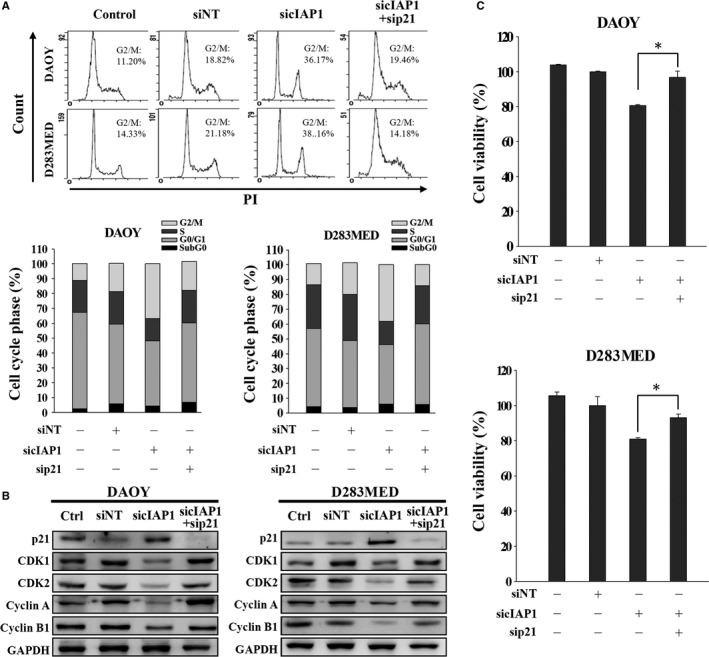
cIAP1 Inhibition Causes G2/M Phase Cell Cycle Arrest Through Upregulation of p21. A, MB cells were transfected with nontargeting (NT) siRNA or siRNA against cIAP1 with or without siRNA against p21 for 72 h. The cell cycle was analyzed by FACS. The proportion of each cell cycle compartment was presented in bar graphs. B, Their total cell lysates were subjected to immunoblotting for detecting cell cycle‐related proteins. C, Cell viability was detected by MTT assay (*P < 0.05)

As p21 expression was upregulated when cIAP1 expression was silenced (Figure 4C), we investigated whether cIAP1‐regulated p21 participates in G2/M transition using FACS. Silencing cIAP1 expression increased p21 and the proportion of G2/M phase and also decreased cell viability in MB cells, yet these effects could be reversed by co‐transfecting with siRNAs against p21 and cIAP1 (Figure 5A,C). Furthermore, ablation of p21 expression can abrogate cIAP1 inhibition‐induced G2/M arrest by retrieving the levels of cyclin A, cyclin B1, CDK1, and CDK2 (Figure 5B). Taken together, these results suggest that inhibiting cIAP1 leads to G2/M phase arrest via upregulation of p21.

### 3.6 LBW242 reduces cIAP1 interaction with p21 and hence abrogates neddylation‐mediated proteasomal degradation of p21

**Figure 6:**
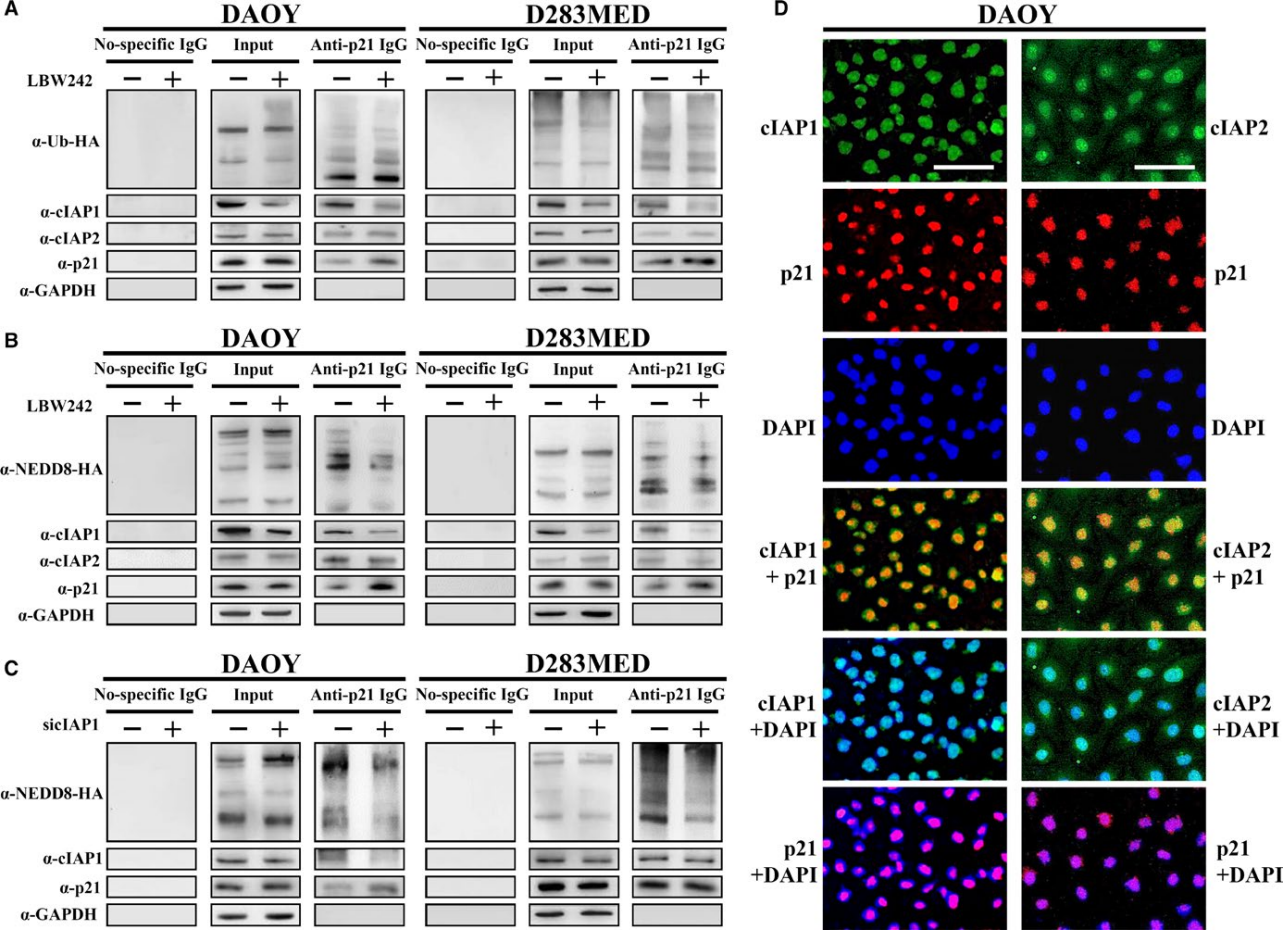
IAP Antagonist LBW242 Attenuates p21‐Interacted cIAP1 Expression and Neddylation of p21. A, To examine the ubiquitination of p21, MB cells were transfected with the plasmid carrying ubiquitin (Ub)‐HA and then treated with LBW242 combined with MG‐132 for 6 h. Total cell lysates were used for immunoprecipitation of p21, and subsequently applied to immunoblotting for detection of Ub‐HA, cIAP1, and cIAP2. B, To detect the neddylation of p21, MB cells were transfected with the plasmid overexpressing NEDD8‐HA, treated with MG‐132 and LBW242, and then subjected to immunoprecipitation. C, To examine whether cIAP1 dominates neddylation of p21, MB cells were co‐transfected with cIAP1 siRNA and NEDD8‐HA overexpressed plasmid, treated with MG‐132 for 6 h, and then applied to immunoprecipitation. D, DAOY cells were treated with MG‐132 for 6 h, and stained by immunofluorescence with anti‐cIAP1, anti‐cIAP2, and anti‐p21 antibodies. The bar scale represents 100 μm

Next, we verified the role of cIAP1 in downregulation of p21 expression. p21 protein can undergo ubiquitination‐mediated proteasomal degradation during cell cycle progression.^32^ Based on this notion, we treated DAOY and D283MED cells with proteasome inhibitor MG‐132 in time course experiments. As expected, their p21 expression was accumulated in a time‐dependent manner. In contrast, treatment with protein synthesis inhibitor cycloheximide (CHX) decreased p21 protein levels after 30 minutes (Figure 4D). These data substantiated that p21 is a bona fide cell cycle regulator degraded through the proteasome system. To further verify whether p21 proteasomal degradation is triggered by ubiquitination, the cells were transfected with a plasmid encoding hemagglutinin‐conjugated ubiquitin (Ub‐HA) and then treated with or without LBW242 in the presence of MG‐132 for 6 hours. Thereafter, p21 in cell lysates was immunoprecipitated using specific antibodies. Nonspecific IgG was a negative control. The levels of ubiquitin interacting with p21 were detected by immunoblotting using anti‐HA antibody. The results indicated that LBW242 had no effect on ubiquitination of p21 (Figure 6A).

According to other studies reporting that IAPs function as E3 ligases for both ubiquitination and neddylation,^32^ we next examined whether LBW242 treatment‐increased p21 protein expression due to abrogation of NEDD8 (neddylation)‐mediated proteasomal degradation. MB cells were treated with NEDD8‐activating enzyme (NAE) inhibitor MLN4924 and their p21 protein levels were determined at different time points. As shown in Figure 4D, p21 protein in both DAOY and D283MED cells accumulated in a time‐dependent manner after treatment with MLN4924. Moreover, MB cell lines transfected with a plasmid carrying NEDD8‐HA for detecting neddylation of p21, then treated with or without LBW242 in the presence of MG‐132 for 6 hours. Immunoprecipitation of p21 showed that LBW242 reduced neddylation of p21 by decreasing its interacted cIAP1 protein levels (Figure 6B).

To investigate whether LBW242 treatment attenuates NEDD8‐mediated proteasomal degradation of p21 through reduction of cIAP1 expression, we knocked down cIAP1 expression using specific siRNA and then treated the MB cells with MG‐132 for 6 hours. Ablation of cIAP1 attenuated the levels of NEDD8‐HA co‐immunoprecipitated with p21 protein (Figure 6C); however, it did not diminish ubiquitination activity of p21 (Figure S5A). Moreover, cIAP2 ablation followed by treatment with MG‐132 did not attenuate ubiquitination or neddylation of p21 (Figure S5B,C). Collectively, our data showed that IAP inhibitor LBW242 particularly reduces cIAP1 expression and consequently interrupts NEDD8‐mediated p21 protein degradation.

### 3.7 There is an inverse relationship between cIAP1 and p21 expression in human MB tumor tissues

**Figure 7:**
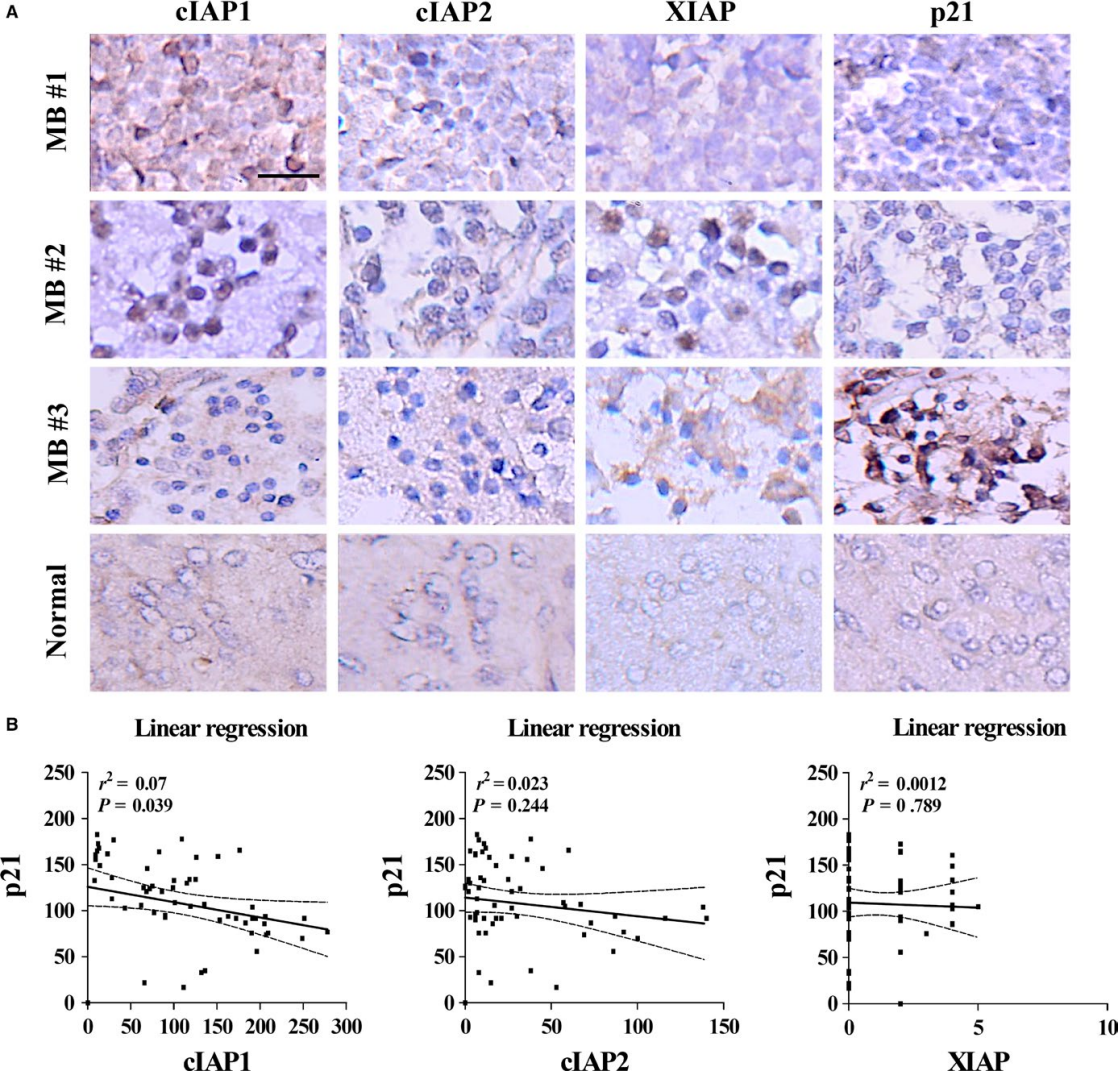
Nuclear cIAP1 Levels Negatively Correlate with Nuclear p21 Levels in MB Tumor Tissues. A, The levels of XIAP, cIAP1, cIAP2, and p21 in MB tissues were detected by IHC staining. The bar scale represents 100 μm. These proteins can be detected in nuclei and cytoplasm. MB tumor tissues possessed higher levels of cIAP1 and cIAP2 and lower levels of p21, whereas normal brain tissues possessed opposite expression of these proteins. B, The levels of these proteins were represented as H‐scores. Linear regression analysis indicated a negative correlation between nuclear cIAP1 and nuclear p21 protein expression in MB tumor tissues (*P *<* *0.05)

As our results indicated that cIAP1 mainly participates in p21 degradation, we examined the correlation between cIAP1 and p21 expression in MB cells and tumor tissues. We observed the localization of cIAP1, cIAP2, and p21 in DAOY cells using immunofluorescence subsequent to treatment with MG‐132. cIAP1 was expressed in the nuclei, while cIAP2 was expressed in the nuclei and slightly present in the cytoplasm (Figure 6D). Although both cIAP1 and cIAP2 colocalized with p21 in the nuclei and co‐immunoprecipitated with p21, only cIAP1 governed p21 degradation (Figure 6A‐D). Moreover, cIAP1, cIAP2, XIAP, and p21 expression in tissue arrays were also detected by IHC staining. As expected, high levels of cIAP1, cIAP2, and XIAP can be seen in 85%, 70%, and 75% of MB tissues, respectively, but their levels were very low in normal brain tissues (Figure 7A). The levels of cIAP1, cIAP2, XIAP, and p21 in nuclei were quantified using H‐scores and their correlations were interpreted using linear regression analysis. There was a significant negative correlation (nonlinear correlation) between nuclear cIAP1 and nuclear p21 expression (*r* ^2^ *= *0.07, *P *=* *0.039), whereas there was no significant inverse correlation between nuclear cIAP2 and nuclear p21 (*r* ^2^ = 0.023, *P *=* *0.244), and between nuclear XIAP and nuclear p21 (*r* ^2^ **=* *0.0012, *P *=* *0.789; Figure 7B).

## 4 DISCUSSION

Although maximal surgical resection of the tumor followed by craniospinal radiotherapy and chemotherapy can improve prognosis, one‐third of MB patients still perish from their disease.^33^, ^34^ Several chemotherapeutic agents including vincristine, cyclophosphamide, lomustine, and cisplatin have been used against these aggressive neoplasms.^34^, ^35^, ^36^, ^37^, ^38^ However, MB cells often develop resistance to traditional chemotherapy and radiation, limiting the therapeutic effectiveness of these cytotoxic drugs.

We previously showed that MB tumors express XIAP and hence treatment with IAP antagonists sensitizes MB cells to conventional chemotherapy, overcoming resistance through induction of apoptosis in CD133+ stem‐like MB cancer cells.^12^ IAPs not only negatively regulate apoptosis by interfering in the caspase cascade but also by other functions.^39^, ^40^ For example, XIAP and cIAP activate NF‐κB and c‐Jun N‐terminal kinases (JNK), resulting in cell survival and proliferation.^21^ Some studies have shown atypical roles for IAPs in chromosome segregation during cell mitosis.^39^, ^41^ Expression of cIAP1 is exclusively present in the cell nuclei of hematopoietic stem cells and some cancer cells. Silencing cIAP1 expression leads to an increase in the G0/G1 phase.^28^ Differing from these results, our data revealed that IAP antagonists and cIAP1/2 siRNAs can slow cell proliferation by inducing G2/M phase arrest, alone or in combination with chemotherapeutic agents (Figures 2A, 3A and Figure S2). Among various cell cycle checkpoint proteins participating in the evolution of the cell cycle, cyclin A‐CDK1 complex and cyclin A‐CDK2 are required for passage into the late S/G2 phase while the cyclin B1‐CDK1 complex is involved in G2/M transition.^42^, ^43^, ^44^ Activity of these CDKs is regulated by inhibitor p21.^45^ Our data showed that inhibition of cIAP1 causes a drastic decrease in cyclin B1‐CDK1 and cyclin A‐CDK1/2 expression but a dramatic increase in their CKI p21 (Figures 2C and 3C). These results are also supported by other studies indicating that expression of IAPs appears in mitotic cells and contributes to cell survival.^25^, ^46^ The discrepancies in cIAP1 inhibition leading to cell cycle arrest in different phases may be due to different cell types and their mitotic activity.

Nuclear cIAP1 overexpression is associated with poor prognosis in bladder cancer, lymph node metastasis and head and neck squamous cell carcinoma patients.^47^, ^48^ We found that nuclear cIAP1 expression is much higher in MB cells relative to normal brain tissues, and inversely correlates nuclear p21 expression (Figure 7). We also found colocalization of cIAP1 and p21 in nuclei of MB cells (Figure 6D). Therefore, nuclear cIAP1 expression may account for the high mitotic activity of MB cells.

At present, there is no literature verifying the mechanism whereby IAPs downregulate p21 expression. According to several studies, upregulation of p21 transcription can be through p53‐dependent pathways or p53‐independent pathways.^49^, ^50^, ^51^, ^52^ The promoter of p21 contains two conserved p53‐binding sites required for p53 responsiveness after DNA damage.^53^ However, XIAP or cIAP1/2 ablation did not affect p53 protein expression (data not shown), and both IAP inhibitors and siRNAs did not alter p21 transcriptional levels (Figures S3 and S4). Thus, p21 protein expression is likely directly governed by IAPs.

Inhibitors of apoptosis proteins have been appreciated to act as E3 ligases for ubiquitin and NEDD8 in many aspects of cell signals including inflammation, cell proliferation, and cell death.^18^, ^23^ It is possible that IAPs participate in regulation of p21 protein stability. Additionally, p21 protein has been reported to undergo degradation via ubiquitin‐ or NEDD8‐proteasome system.^32^ Hence, we investigated whether p21 undergoes ubiquitin‐dependent proteasomal degradation. Similar to MG‐132 treatment, IAP inhibitor LBW242 can enhance accumulation of p21 protein in a time‐dependent manner (Figure 4A,B). Silencing cIAP1 also increased protein levels of p21 (Figure 4C). Surprisingly, neither treatment with LBW242 nor cIAP ablation reduced ubiquitination of p21 (Figure 6A and Figure S5A,B). Therefore, IAPs‐induced p21 protein degradation is not mediated by ubiquitin. NEDD8 conceivably takes over p21 proteasomal degradation.

Neddylation is the process where NEDD8 is conjugated to target substrates by NEDD8‐activating enzyme (NAE), NEDD8‐conjugating enzyme E2, and substrate‐specific E3s in diverse processes, such as transcription, signal transduction, cell cycle progression. One well‐known inhibitor targeting NAE, MLN4924, is known to suppress cell growth by inducing p21‐dependent S and G2/M phase arrest in several cancers.^54^, ^55^, ^56^, ^57^, ^58^ Therefore, this study used LNM4924 to confirm neddylation of p21 (Figure 4D). Immunoprecipitation analysis showed that less NEDD8 can be pulled down with p21 protein when treated with LBW242 or silencing cIAP1 (Figure 6B,C). Taken together, these data suggest that cIAP1 initiates p21 protein degradation through activation of the neddylation(NEDD8)‐proteasome system. This is our novel finding uncovering a new role of cIAP1 in p21 protein degradation.

**Figure 8:**
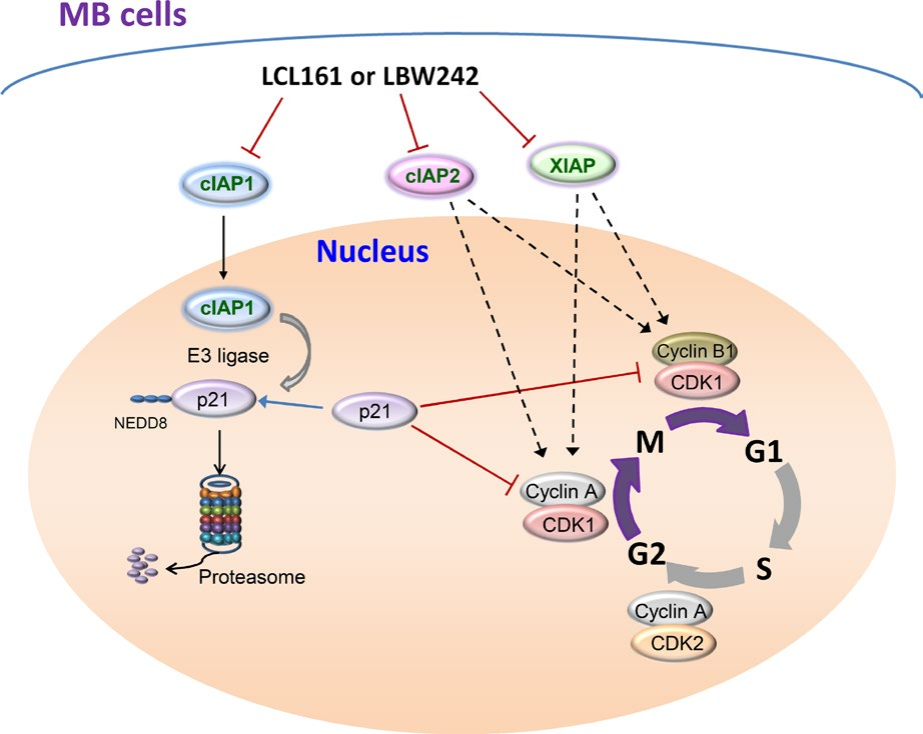
The Schematic Diagram Elucidates the Mechanism Whereby IAP Antagonists Induce G2/M Phase Arrest in MB Cells. IAP antagonists LBW242 and LCL161 induce MB cell cycle arrest in the G2/M phase through decreased expression of cIAP1, cIAP2, and XIAP. G2/M phase arrest is attributed to attenuation of cyclin A‐CDK1/2 and cyclin B‐CDK1. Downregulated cIAP1 expression fails to induce neddylation (NEDD8)‐mediated proteasomal degradation of p21, resulting in elevation of p21 protein levels which consequently disturbs the cell cycle in MB cells

In summary, our data present novel insights regarding the effect of IAPs on regulation of the cell cycle: (a) inhibition of cIAP1/2 or XIAP in combination with conventional chemotherapy results in G2/M phase accumulation in MB cells; (b) silencing cIAP1 expression leads to upregulation of p21 and subsequent suppression of cyclin B1‐CDK1 and cyclin A‐CDK1/2; (c) cIAP1 can degrade p21 protein through activation of the NEDD8‐proteasome system; (d) IAP inhibitors attenuate cIAP1‐induced neddylation of p21; (e) nuclear cIAP1 expression negatively correlates with nuclear p21 expression in MB tumor tissues. These mechanisms have been illustrated in Figure 8.

## CONFLICT OF INTEREST

The authors have no conflict of interest.
