# Supplementary material for: Targeting inhibitors of apoptosis proteins suppresses medulloblastoma cell proliferation via G2/M phase arrest and attenuated neddylation of p21
Source: Cancer Med. 2018 Jul 9;7(8):3988–4003. doi: 10.1002/cam4.1658 (PMC6089189; doi:10.1002/cam4.1658)
Supplement: Supplementary file 1 [file CAM4-7-3988-s001.pdf]

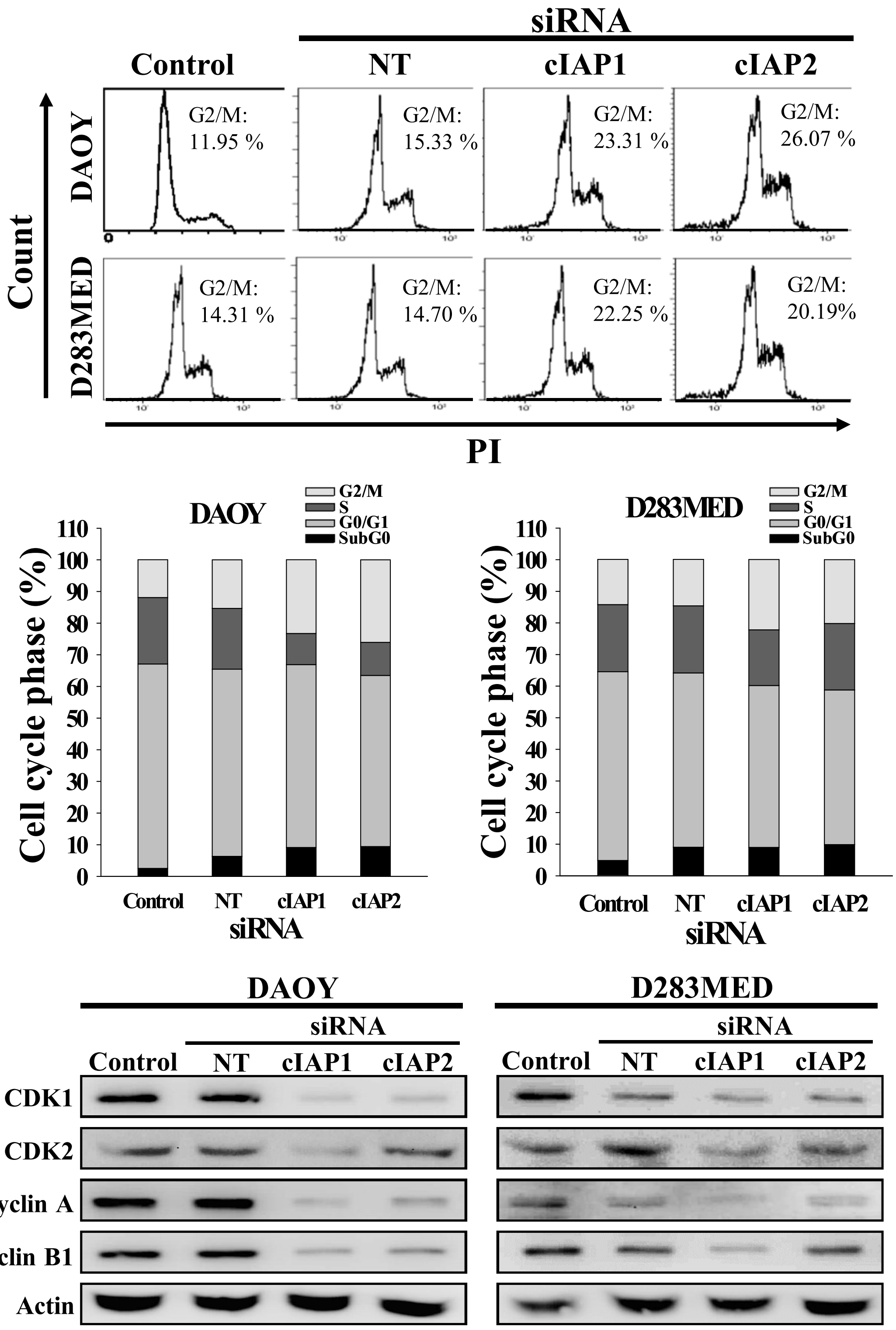

Fig. S1. Knockdown of cIAP1 or cIAP2 disturbs the cell cycle in DAOY and D283MED cells.

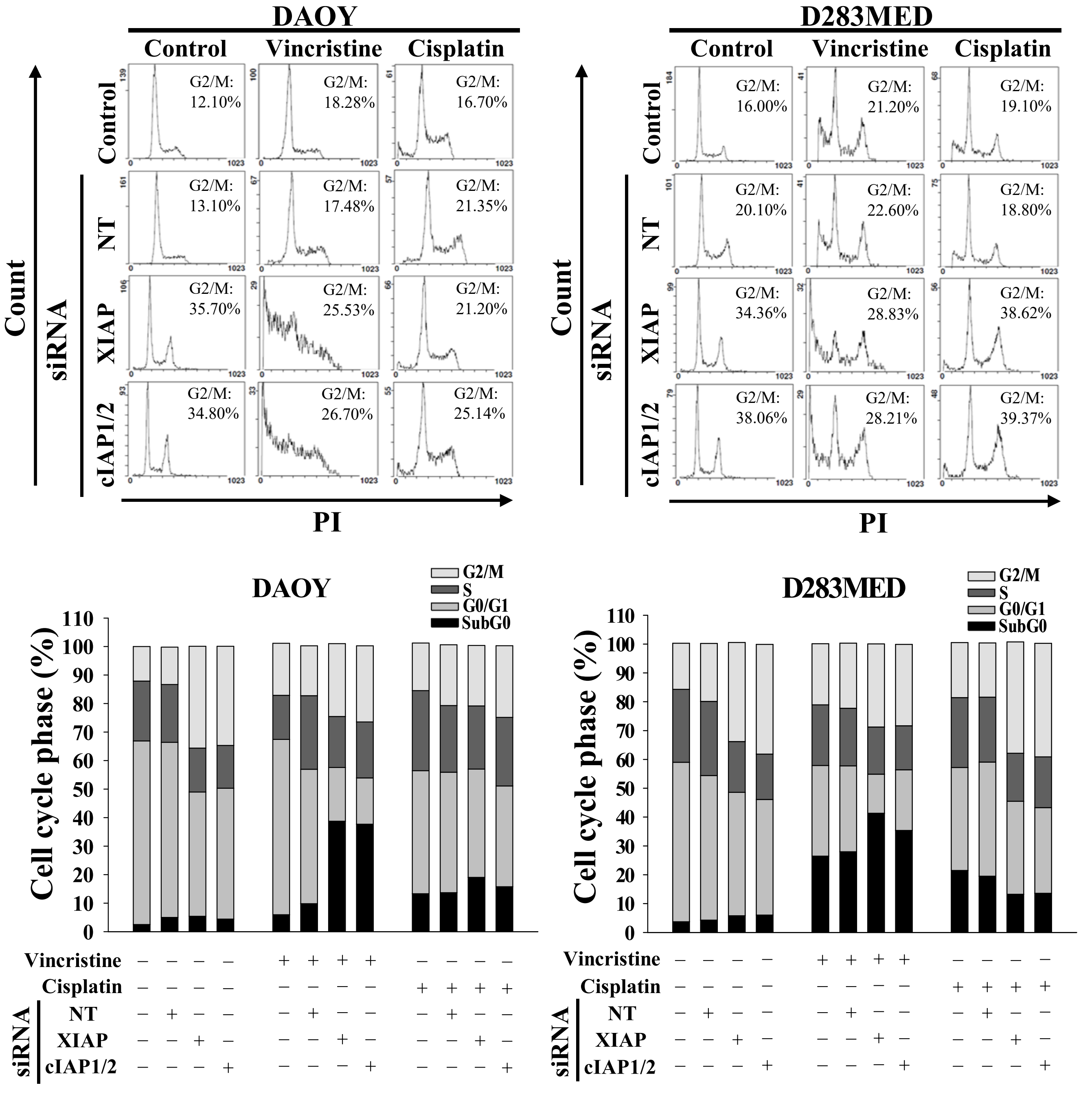

Fig. S2. Knockdown of cIAP1 or cIAP2 or in combination with DMSO control or chemotherapeutic agent (vincristine or cisplatin) alters cell cycle in MB cells.

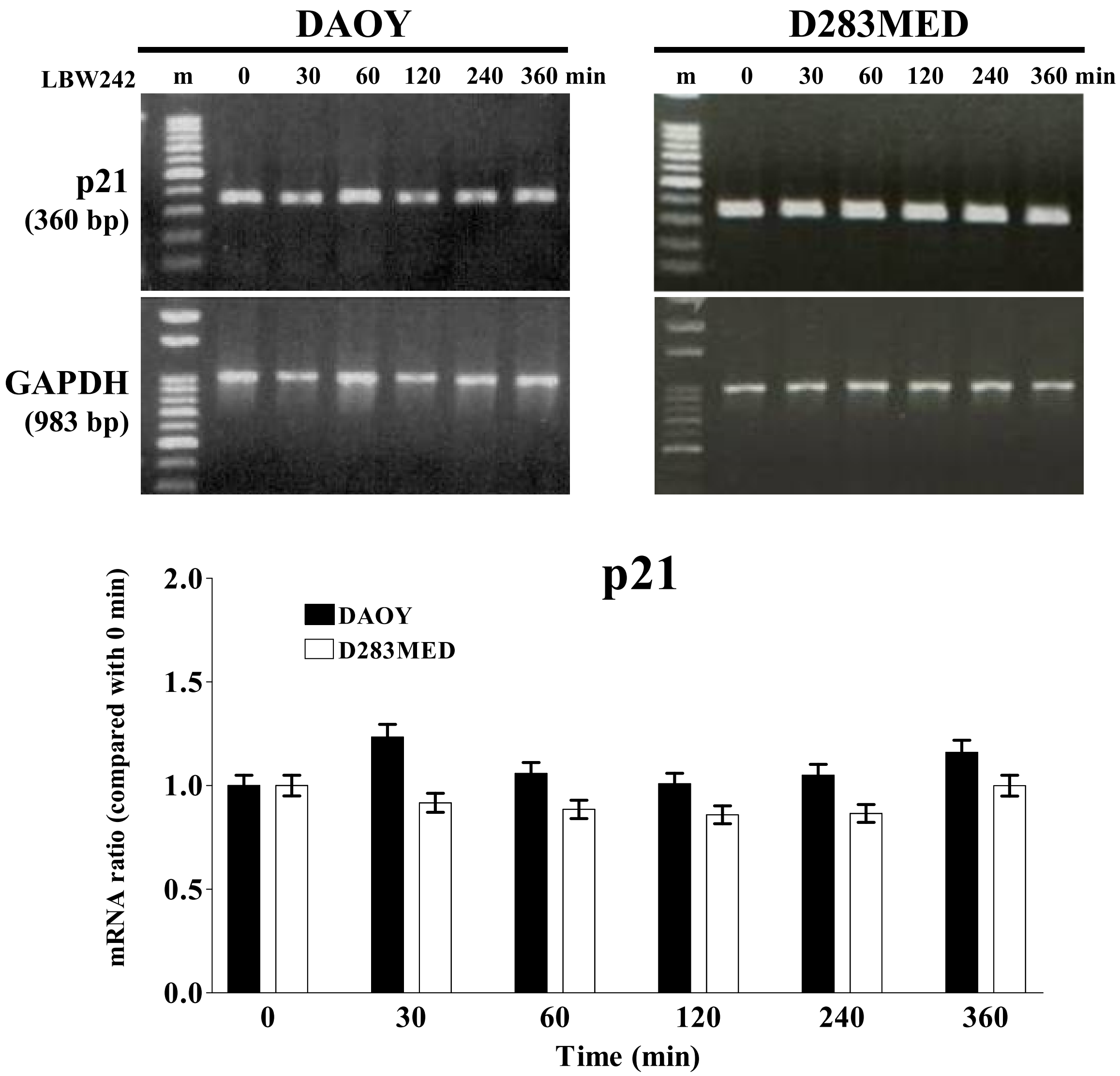

Fig. S3. RT-PCR analysis reveals that IAP inhibitor LBW242 does not affect p21 RNA levels in MB cells.

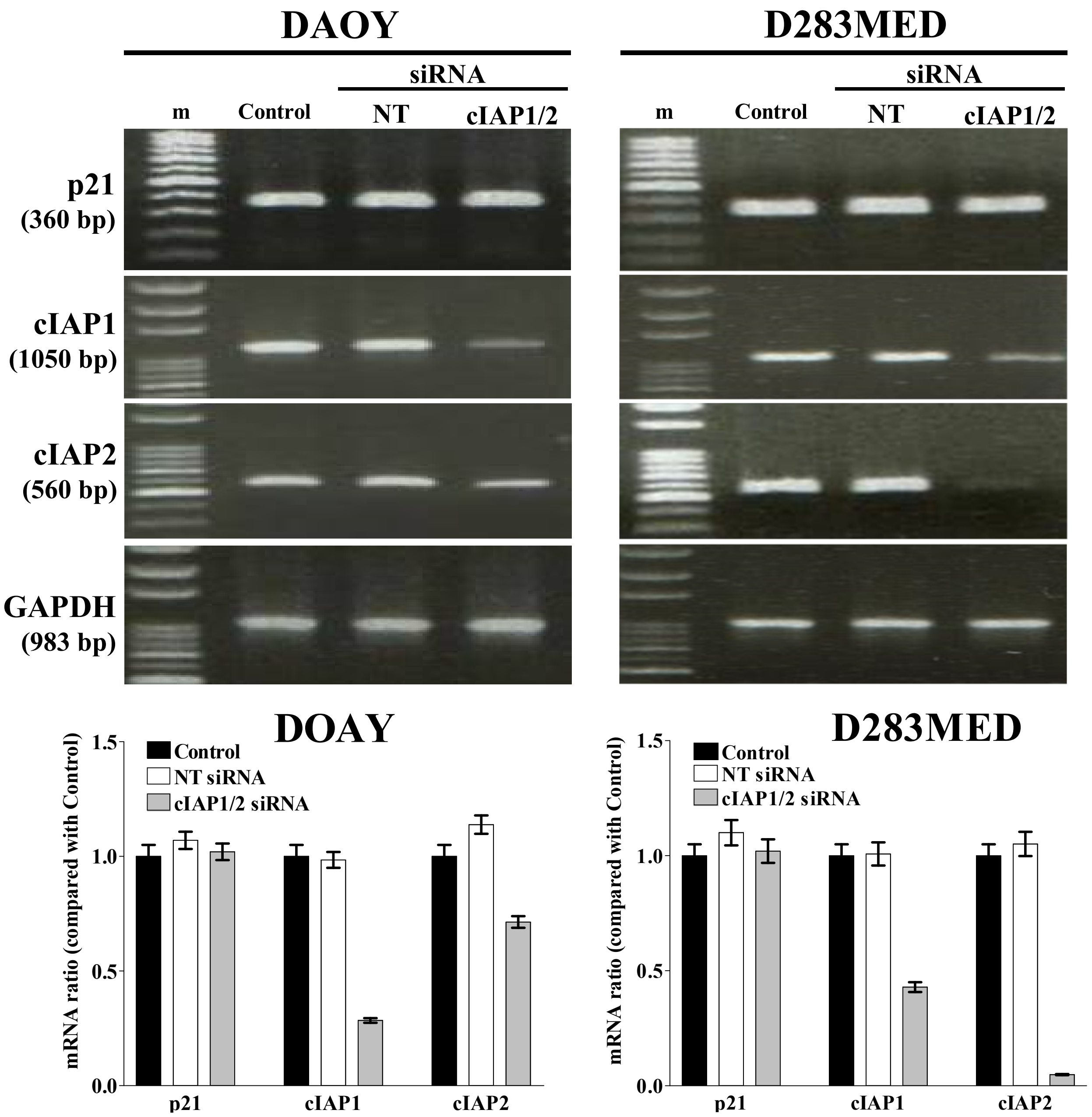

Fig. S4. RT-PCR analysis demonstrates that cIAP1/2 ablation using specific siRNA cannot increase RNA levels of p21 in DAOY and D283MED cells.

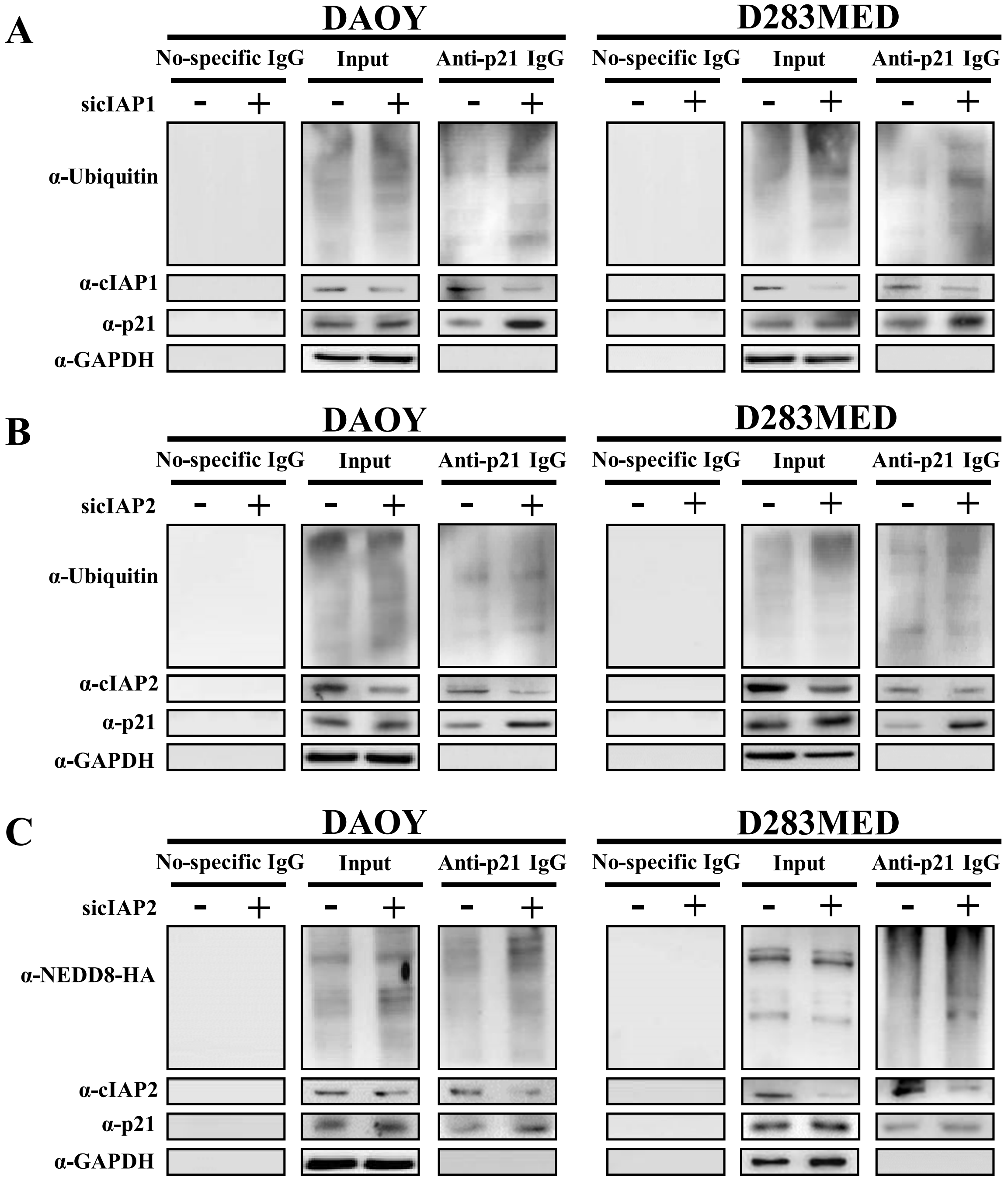

Fig. S5. Silencing cIAP1 or cIAP2 expression cannot reduce ubiquitination of p21 (A), and silencing cIAP2 is unable to reduce neddylation of p21 (B).
